# Massive cardiomegaly secondary to rheumatic heart disease

**DOI:** 10.11604/pamj.2025.50.32.46457

**Published:** 2025-01-24

**Authors:** Gaurang Aurangabadkar, Sumer Choudhary

**Affiliations:** 1 Department of Respiratory Medicine, Datta Meghe Medical College, Nagpur, Datta Meghe Institute of Higher Education and Research (DMIHER), (Deemed University), Sawangi (Meghe), Wardha, Maharashtra, India

**Keywords:** Cardiomegaly, rheumatic heart disease, mitral valve stenosis

## Image in medicine

A 54-year-old female patient presented to the respiratory physician with chief complaints of dyspnea on exertion, dysphagia, and chest pain. The patient´s past medical history revealed a diagnosis of rheumatic heart disease, which was initially diagnosed 8 years back and a recent echocardiography report was suggestive of severe mitral stenosis with a left ventricular ejection fraction (LVEF) of 28%. An esophagoscopy was done given dysphagia which revealed no obvious abnormalities of the esophageal mucosa. A chest X-ray postero-anterior (PA) view was done which revealed the presence of a massive cardiomegaly with a cardiothoracic ratio of 0.80 (normal cardio-thoracic ratio <0.50). A cardiologist's opinion was taken and the patient was started on angiotensin-converting enzyme (ACE) inhibitors, oral furosemide (diuretic), and carvedilol (beta-blockers), along with regular follow-up. The patient was discharged with the same advice after 5 days of admission. Gross cardiomegaly is a rare complication of rheumatic heart disease, usually seen in patients with severe mitral stenosis, and occurs as a result of altered cardio-pulmonary hemodynamics arising as a result of valvular pathology. Such patients usually present with complaints of dyspnea and dysphagia arising as a result of the considerable enlargement of the cardiac dimensions. This clinical image aims to highlight this striking presentation of gross cardiomegaly that is seen to occupy more than 75% of the hemithorax in horizontal dimensions.

**Figure 1:**
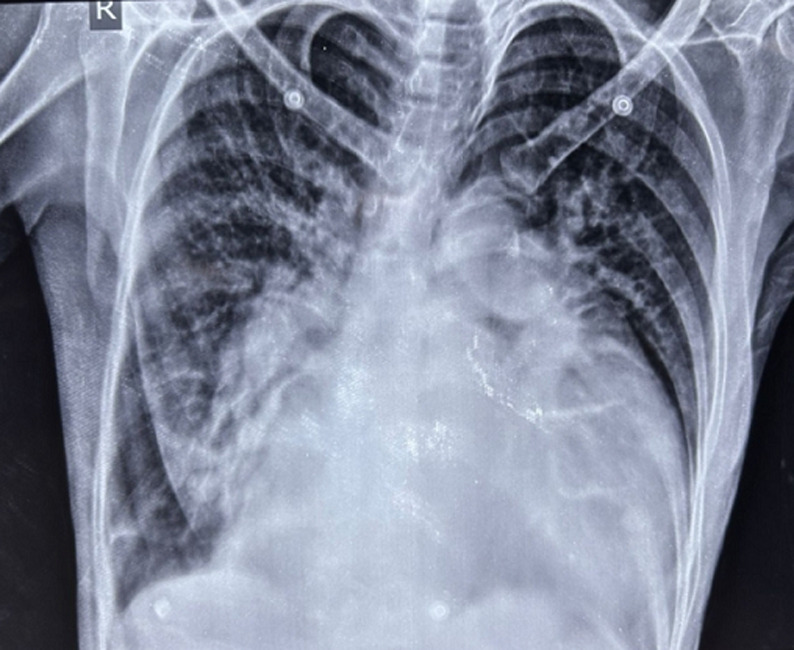
chest X-ray postero-anterior (PA) view demonstrating gross cardiomegaly in a patient with rheumatic heart disease

